# Integrative miRNA-Gene Expression Analysis Enables Refinement of Associated Biology and Prediction of Response to Cetuximab in Head and Neck Squamous Cell Cancer

**DOI:** 10.3390/genes8010035

**Published:** 2017-01-14

**Authors:** Loris De Cecco, Marco Giannoccaro, Edoardo Marchesi, Paolo Bossi, Federica Favales, Laura D. Locati, Lisa Licitra, Silvana Pilotti, Silvana Canevari

**Affiliations:** 1 Functional Genomics and Bioinformatics, Department of Experimental Oncology and Molecular Medicine, Fondazione IRCCS Istituto Nazionale dei Tumori, Milan 20133, Italy; marco.giannoccaro@istitutotumori.mi.it (M.G.); edoardo.marchesi@istitutotumori.mi.it (E.M.); 2 Head and Neck Medical Oncology Unit, Fondazione IRCCS Istituto Nazionale dei Tumori, Milan 20133, Italy; paolo.bossi@istitutotumori.mi.it (P.B.); federica.favales@istitutotumori.mi.it (F.F.); laura.locati@istitutotumori.mi.it (L.D.L.); lisa.licitra@istitutotumori.mi.it (L.L.); 3 Laboratory of Experimental Molecular Pathology, Department of Diagnostic Pathology and Laboratory, Fondazione IRCCS Istituto Nazionale dei Tumori, Milan 20133, Italy; silvana.pilotti@istitutotumori.mi.it

**Keywords:** miRNA, microarray, head and neck squamous cell carcinomas (HNSCC), cetuximab, drug sensitivity

## Abstract

This paper documents the process by which we, through gene and miRNA expression profiling of the same samples of head and neck squamous cell carcinomas (HNSCC) and an integrative miRNA-mRNA expression analysis, were able to identify candidate biomarkers of progression-free survival (PFS) in patients treated with cetuximab-based approaches. Through sparse partial least square–discriminant analysis (sPLS-DA) and supervised analysis, 36 miRNAs were identified in two components that clearly separated long- and short-PFS patients. Gene set enrichment analysis identified a significant correlation between the miRNA first-component and EGFR signaling, keratinocyte differentiation, and p53. Another significant correlation was identified between the second component and RAS, NOTCH, immune/inflammatory response, epithelial–mesenchymal transition (EMT), and angiogenesis pathways. Regularized canonical correlation analysis of sPLS-DA miRNA and gene data combined with the MAGIA2 web-tool highlighted 16 miRNAs and 84 genes that were interconnected in a total of 245 interactions. After feature selection by a smoothed t-statistic support vector machine, we identified three miRNAs and five genes in the miRNA-gene network whose expression result was the most relevant in predicting PFS (Area Under the Curve, AUC = 0.992). Overall, using a well-defined clinical setting and up-to-date bioinformatics tools, we are able to give the proof of principle that an integrative miRNA-mRNA expression could greatly contribute to the refinement of the biology behind a predictive model.

## 1. Introduction

Head and neck cancers develop in the mucosal linings of the upper aerodigestive tract and over 90% are squamous cell carcinomas (HNSCC). The disease is diagnosed in advanced stages (stage III and IV) in the majority of patients and their treatment usually requires surgery, radiation therapy, or chemotherapy in different combinations [1]. However, 27%–50% of cases relapse within two years after treatment, and platinum-based chemotherapy (CT) plus a targeted therapy with an anti-EGFR (epidermal growth factor receptor) monoclonal antibody (mAb), cetuximab, is usually offered to recurrent or metastatic (RM) patients.

This systemic treatment still represents that achieving major improvement in RM-HNSCC patients in the last decade [2]. Since only about 40% of patients benefit from a response to this drug combination, and only a small portion of this group experience durable responses, clinicians are faced with the problem of choosing the best curative treatment without the help of reliable predictive biomarkers.

In the last 15 years we have observed continuous improvements in genomics, enabling the molecular profiling of over 20,000 genes and 2000 microRNAs (miRNAs). Similarly, there have been improvements in bioinformatics through the continuous development of new algorithms to integrate these two types of highly dimensional “omics” data [3].

Recent work has shown that by analyzing gene expression profiling of a selected retrospective series of HNSCC patients, and by associating it to the response/sensitivity to cetuximab-CT, we were able to propose that long-progression-free survival (PFS) cases behave in a manner consistent with a defined molecular subgroup. This subgroup has a poor prognosis, which can be rescued by cetuximab/CT treatment, while short-PFS cases are characterized by an over-activation of RAS signaling [4].

Taking advantage of the same HNSCC clinical materials, and on the basis of our successful analyses of other cancer types for biological characterization [5] or for prognostication [6], we profiled miRNAs and we performed an integrated miRNA-mRNA expression analysis to further gain insight into the biology and prediction of the cetuximab-CT response.

## 2. Materials and Methods

### 2.1. Patients and Study Design

Forty tumor specimens from RM-HNSCC patients were divided according to PFS following cetuximab-CT treatment in 14 long-PFS patients and 26 short-PFS patients [4]. The study design includes two groups balanced for known prognostic factors [7] (primary tumor site, performance status, weight loss, prior radiotherapy, tumor grade, residual disease at primary tumor site, age, and gender). Long-PFS had a median PFS of 19 months (range 12–36), while short-PFS had a median PFS of three months (range 1–5.5). The study was conducted in accordance with the Declaration of Helsinki and was approved by the Independent Ethics Committee of Fondazione IRCCS Istituto Nazionale dei Tumori (Approval Number 55/12).

### 2.2. miRNA Profiling

Total RNA was extracted from formalin fixed paraffin embedded (FFPE) tissues using the miRNeasy FFPE kit (Qiagen, Valencia, CA, USA), and concentration was assessed with the NanoDrop ND-100 Spectrophotometer (NanoDrop Technologies, Wilmington, DE, USA). RNA was processed for miRNA profiling according to the manufacturer′s recommendations, and miRNA expression analysis was performed using SurePrint G3 Human miRNA 8×60K microarrays from Agilent Technologies (Santa Clara, CA, USA). RNA was dephosphorylated in the amount of 100 ng with calf intestinal alkaline phosphatase and denatured by DMSO treatment. Samples were labeled with cyanine 3-pCp using T4 RNA ligase and hybridized on miRNA array. Arrays were washed in Agilent′s Wash Buffers and scanned at a resolution of 2 mm using an Agilent DNA microarray scanner (Agilent Technologies). Primary data were collected using Agilent′s Feature Extraction software v10.7 (Agilent Technologies). Raw miRNA expression data were processed using an optimized version of the Robust Multi-array Average (RMA) algorithm implemented in AgiMicroRna package [8]. Based on gIsGeneDetected information provided by the Agilent′s Feature Extraction software, miRNAs detected in at least 10% of samples were selected, yielding a data matrix containing 614 miRNAs. Microarray data were deposited and are available on NCBI Gene Expression Omnibus (GEO) database [9] with the accession number GSE92595.

### 2.3. Statistical and Bioinformatics Analyses

#### 2.3.1. miRNA Expression Analysis

To identify expression patterns related to patient′s PFS, we applied two different approaches: (i) discriminant analysis aimed at identifying features whose expression differences help stratifying patients; (ii) differential expression analysis aimed at obtaining a list of features whose expression differs among classes. In general, a discriminant feature is differentially expressed, but the reverse is not always true. Among discriminant methods, we used the sparse partial least square–discriminant analysis (sPLS-DA) [10] applying mixOmics R package [11] from Bioconductor [12], since in comparative studies it resulted in efficient generalization ability [13]. sPLS-DA is a pattern recognition technique that enables disclosing a set of features in a supervised classification context that are summarized in appropriate linear combinations performing variable selection and classification in a single step procedure. This approach has already been successfully applied to miRNA analysis in a setting where the number of samples is limited compared to the number of tested miRNAs [14]. The discriminative miRNAs were selected on the miRNA data matrix named *X* (*n* × *p*), where *n* is the number of samples and *p* is the total number of variables (i.e., miRNAs). In our analysis, the size is *n* = 40 samples and *p* = 614 miRNAs. The selection was based on the response dummy matrix partitioned in *K* groups (*K* = 2), where *K* is the number of classes. Two parameters should be tuned in sPLS-DA: the number of discriminant vectors and the number of variables to select on each dimension (PLS component). According to further literature [10], it is conventional to choose the number of sPLS-DA dimensions H  ≤  min (*p*, *K*). We tested the performance in terms of the classification error rate with a maximum distances prediction method for the first 10 sPLS-DA dimensions, which included a number of miRNAs ranging from 1 to 50 in each dimension. A 10-fold cross-validation setting was imposed and the “optimal” number of variables was finally determined when the lowest error rate is obtained. Differential expression analysis, imposing the same criteria of Bossi et al. [4], was performed on miRNA expression data, and through a random variance *t*-test that improved estimates without assuming that all miRNA have the same variance. A False Discovery Rate (FDR, [15]) correction was applied on raw *p*-values and a threshold of 0.15 was set. A global test to ascertain the differences in expression patterns between classes was performed through random permutations of the class labels. For each random permutation, all *t*-tests are re-computed for each miRNA and the proportion of the random permutations that gave as many miRNA significant at FDR < 0.15 as were found in comparing the true class labels was assessed providing a *p*-value. BRB-ArrayTools (version 4.3.1) developed by Dr. Richard Simon and the BRB-ArrayTools Development Team, available at [16] was used for differential expression analysis.

#### 2.3.2. Inference of miRNA Components on Gene Expression Data by GSEA

Pathway enrichment analysis was carried out by gene set enrichment analysis (GSEA) using the dataset GSE65021 [4] and the GSEA 2.2.2 software [17]. The first and second components of miRNA sPLS-DA defined the two continuous traits to which gene-expression data were ranked through GSEA, determining gene-sets having either positive or negative correlations. Pearson correlation was used as the metrics for ranking genes, and phenotype permutation was performed to assess the significance of the enrichment scores. For multiple testing adjustments, Benjamini and Hochberg′s false discovery rate was applied. FDRs were calculated on the normalized enrichment scores (NES) and only gene-sets enriched with an FDR < 0.05 were retained. GSEA was performed on Molecular Signatures Database (MSigDB; v5.1 updated January 2016, http://software.broadinstitute.org/gsea/msigdb), including C5 collection associated with GO terms, C6 oncogenic signatures corresponding to cellular pathways dis-regulated in cancer by specific oncogenes, and Hallmark, a collection of gene-sets aggregating many MSigDB redundant terms to represent well-defined biological processes. Graphical representation of the most significant gene-sets was provided by GOBubble function available in GOplot R package [18], displaying information about the significance of the enrichment (−log10 *p*-value) and the z-score of each gene set. 

#### 2.3.3. Differential Gene Expression Analysis by sPLS-DA

The procedure described in Section 2.3.1. for miRNA expression data was also applied on gene-expression data corresponding to a data matrix retrieved from GEO repository under the ID GSE65021 [4] denominated *Z* (*n* × *q*) whose size is 40 (samples) × 17,378 (detected features = genes).

#### 2.3.4. miRNA and Gene-Expression Integrative Analysis

We focused our attention on analytic methods capable of establishing the potential relationships between sets of measurements from the same samples, reducing the dimensionality of the data. Canonical correlation analysis (CCA) is a classical technique developed by Hotelling [19] that extracts linear correlations among sets of variables on the same set of subjects, including all variables from both data sets. However, limited sample size and high dimensional data, which is a common situation for genomic studies, results in inaccurate estimates and data overfitting issues [20]. Regularized CCA (rCCA) is a modified version of CCA that implements a regularization procedure to reduce the dimensionality of the data. This method has been already used [21] and resulted useful to disclose linear relationships between two sets of variables. Taking into account two data matrices *X* (*n* × *p*) and *Z* (*n* × *q*), representing miRNA and gene-expression data, respectively, where *n* << *p* and *n* << *q*, regularization of covariance′s matrices of *X* and *Z* consists in adding a multiple of the identity matrix (Id):

Cov(*X*) + λ_1_Id and Cov(*Z*) + λ_2_Id

The regularization parameters λ_1_ and λ_2_ were selected by 10-fold cross-validation procedure on a regular grid of size 100 × 100 defined on the region 0.001 < λ_1_ < 0.05, and 0.0001 < λ_2_ < 0.05 using the *tune.rcc* function available in mixOmics and resulting in λ_1_ = 0.0292 and λ_2_ = 0.00199 (Supplementary Materials Figure S1A). The choice of dimensions *d* (1 ≤ *d* ≤ *p*) to include in further analysis was done according to Gonzalez et al. [22] who suggests an empirical approach based on the inspection of the plot of canonical correlations versus dimensions and on selection of the appropriate number of dimensions before a clear gap among canonical correlations. On the basis of the obtained λ_1_ and λ_2_ parameters, we observed a clear gap between the 23rd and the 24th canonical correlations (Supplementary Materials Figure S1B).

The relationships between miRNAs and genes were displayed by correlation circle plots that allow recognition of the correlation structure between the two sets of variables *X* and *Z*. In this type of graphical representation, the variables *X* and *Z* are projected as vectors onto a plane and the relationship (correlation) between variables is approximated by the inner product between vectors (i.e., the product of the two vector lengths and their cosine angles). Since the variables *X* and *Z* correspond to unit of variance, their projections are inside a circle of radius 1 centered at the origin of the correlation plot. In this way, the correlation circle plots allow to reveal inherent features embedded into the correlation structure of the variables. In detail, the association among variables is disclosed by: (i) the distance from the origin (the greater the distance is, the stronger the association is); (ii) the direction of the projection of variables from the origin (sharp angle in the same direction meaning a positive correlation; obtuse angle in an opposite direction meaning a negative correlation; a right angle meaning null correlation). To improve interpretability, two circles are drawn: one external circle, radius = 1, and one internal circle. The area between the two circles reveals the most important variables and the variables close to the center are assumed to be not relevant. By imposing in our analysis an inner circle with radius = 0.3, the area from 0.3 to 1 contains the relevant miRNA-gene expression relationships. rCCA results were also displayed by clustered image maps (CIM) as a heatmap that represents the correlation between two matched datasets. The similarity matrix obtained by miRNA and gene-expression was organized in a bi-cluster hierarchical structure illustrating the interplay among sets of variables [23]. The Euclidian distance and the average agglomeration method were used for the hierarchical clustering.

#### 2.3.5. Target Prediction

We integrated target predictions with correlation-based miRNA and gene expression profiles based on the hypothesis that the expression profile of a given miRNA is expected to be inversely correlated with its mRNA target if the miRNA acts on mRNA stability. Data from miRNA and gene expression identified by rCCA were analyzed by MAGIA2 web-tool [24] to build mixed miRNA-gene expression networks. MAGIA2 was run on the expression data for the top 75% of genes with greatest variation in expression among samples. Three target prediction databases, DIANA-microT [25], TargetScan [26], and microrna.org [27], were selected for miRNA target prediction using default settings. The predictions shared by at least two of the three databases are retained and the final network is visualized by Cytoscape [28]. To investigate the experimentally validated miRNA/gene interactions, we retrieved the data from miRTarBase, a comprehensive repository of evidences based on literature [29].

#### 2.3.6. miRNA-Gene Integrative Predictive Signature

To test the feasibility in defining a miRNA-gene integrative predictive signature, a method is required satisfying the criteria to be able to: (i) select pivotal features avoiding unnecessary redundancy; (ii) integrate data from different sources (i.e., miRNA and gene expression). Smoothed *t*-statistic support vector machine (stSVM) is a feature selection method that proved its efficiency in merging network data by smoothing feature-wise *t*-statistics using a random walk kernel and the integration of data from miRNA and gene expression profiles [30]. The method was designed to select features by a permutation test with subsequent SVM training. Prediction performance was assessed by 10-time repeated 10-fold cross-validation. All calculations were performed through *netClass* R-package [31].

#### 2.3.7. Comparison Analysis with Publically Available Data

Level 3 files of HNSCC miRNA and gene expression data present in The Cancer Genome Atlas (TCGA) [32] were downloaded along with the clinical annotations from the TCGA website [33] and used for the analysis. Subtype molecular classification was performed as described in De Cecco et al. [34] identifying Cl2-mesenchymalmesenchymal and Cl3-hypoxia subtypes.

## 3. Results

### 3.1. miRNA Expression Patterns in HNSCC Patients Treated with Cetuximab-CT

**Figure 1:**
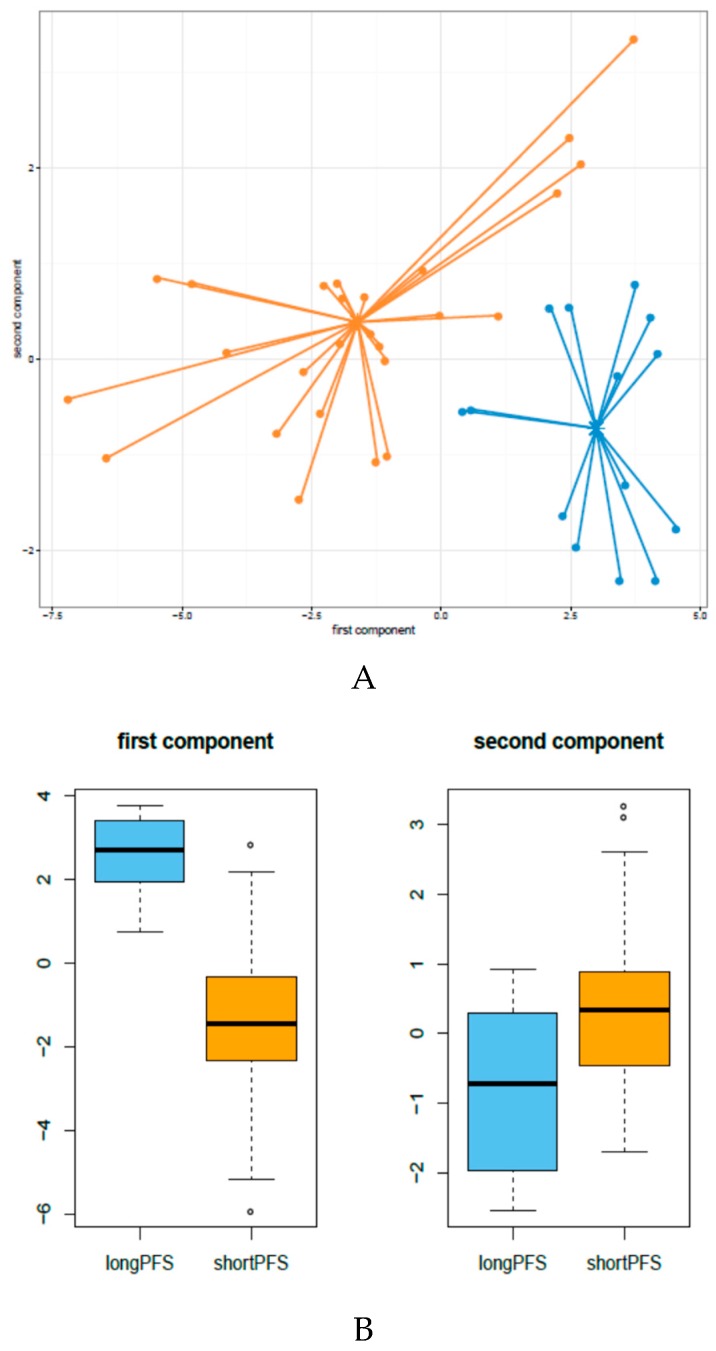
miRNA expression patterns in HNSCC tumors associated with PFS after cetuximab-CT treatment: sPLS-DA identified 24 and 12 miRNAs in first and second components, respectively (see Supplementary Materials Table S1 for list). (**A**) The score plot of sPLS-DA miRNA profiles of the two components is shown and each individual patient was plotted. The well-defined clusters, corresponding to the 14 long-PFS patients (blue dots) and the 26 short-PFS patients (orange dots), can be identified. The lines indicate the distance from the respective centroid for samples of each class; (**B**) The box-plot analysis shows the loading vector values for the first component and second component with samples divided on the basis of class (long PFS = blue; short PFS = orange). A significant difference was observed in each component (first component, *p* = 2.97E-07; second component, *p* = 0.00529).

To disclose miRNA patterns associated with PFS, a supervised analysis was applied to our data matrix of 614 detected miRNAs using sPLS-DA and imposing the “a priori” knowledge of a patient′s PFS category (long and short PFS) [4]. sPLS-DA allows selection for variables that best separate the two PFS categories, reducing the dimensionality of those patterns in a few discriminant components. We assessed the classification error rate using the maximum distance method to determine the optimal number of dimensions and features to retain. As such, the best performance (lowest misclassification rate) was obtained, including the first two components which explained 68% of the variance in miRNA expression; the first component (24 miRNAs), which retained a large part of the variance, explained 49% of the variance, compared to the second component (12 miRNAs), which explained 19% of the variance (miRNA list in Supplementary Materials Table S1, panel A). The results of this analysis showed that miRNA profiles were able to clearly separate long- and short-PFS patients (Figure 1A) and each component significantly divided the two categories of patients (Figure 1B).

When the same data matrix was analyzed by supervised class comparison, we identified 166 differentially expressed miRNAs (the probability of finding 166 miRNAs significant by chance was 0.013, as determined by the global test). After filtering based on |log_2_(fold change)| > 1, 39 miRNAs, four and 35 upregulated in long- and in short-PFS samples, respectively, were retained (volcano plot in Supplementary Materials Figure S2; fold change and *p*-values in Supplementary Materials Table S1, panel B). The comparison of the two methods (see Supplementary Materials Table S1) indicated that 18 of 39 differentially expressed miRNAs were included in the first component of the sPLS-DA while none from the second component were differentially expressed according to the selection criteria.

### 3.2. Biological Relevance of sPLS-DA miRNAs Inferred by GSEA

**Figure 2:**
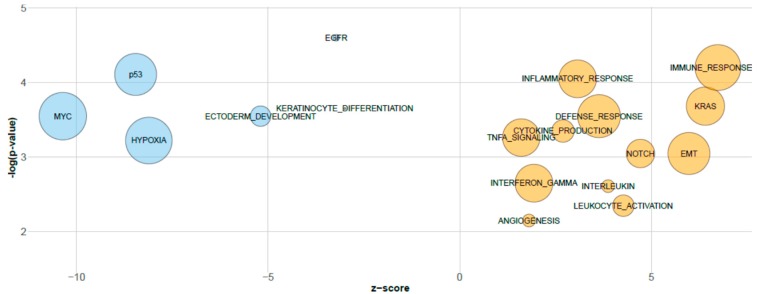
Bubble plot of gene sets associated with miRNAs’ first and second components identified by sPLS-DA: An overview of GSEA-enriched networks was depicted. The x-axis indicates the z-score for each term, while the y-axis is the negative logarithm of the adjusted *p*-value. The area of the displayed circles is proportional to the number of genes assigned to each term. The colors display the positive association to the first (blue) and second (orange) components.

In order to investigate the biological information embedded in the miRNA sPLS-DA components, these components were correlated to gene expression data [4] and pathway recognition was performed through GSEA. miRNAs of the first component correlated with EGFR signaling, keratinocyte differentiation and ectoderm development (GO terms), and p53 and Myc (oncogenic signatures); miRNAs of the second component were correlated with the oncogenic signatures RAS and NOTCH and with numerous GO terms, including immune/inflammatory response, EMT, and angiogenesis pathways (Figure 2).

### 3.3. Integrated miRNA and mRNA Networks

**Figure 3:**
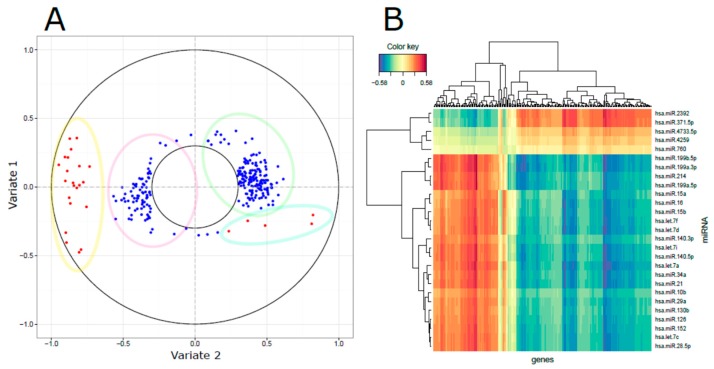
Identification of integrated miRNA and mRNA networks: (**A**) The correlation circle plot displays the features (miRNAs = red; genes = blue) and the position derived by the combination of the first and second variates in our rCCA. The features in the area outside the inner ring (radius < 0.3) were retained as significant and shown in the plot. The ellipses depicted four miRNA/gene clusters and the list can be found in Supplementary Materials Table S2; (**B**) The CIM plot displays the correlation structure between the features. Each colored block represents an association between miRNAs and genes spanning a range of colors from blue (negative correlation), to yellow (weak correlation), to red (positive correlation). The dendrograms on the top and left side indicate how genes and miRNAs, respectively, are connected.

To proceed in an integrative analysis, we first applied sPLS-DA on whole-gene expression data obtained from the same samples (see Bossi et al. for details; [4]) and following the criteria of the miRNA profile. A lower misclassification rate was found including the first two components containing 428 unique genes; Supplementary Materials Figure S3 shows the performance in stratifying patients based on long and short PFS. Through rCCA we tested the linear combinations between *X* and *Z* data matrices, representing miRNA and gene expression patterns, respectively; rCCA allowed us to highlight the most relevant correlation structures embedded into the expression data. Since the first two variates explained most of the data variability (45% and 14% variates 1 and 2, respectively) and retained the ability to stratify patient outcome, we restricted the analysis to these dimensions to identify relevant miRNA–gene expression associations. The results of the integrated analysis are displayed through a correlation circle plot (Figure 3A) in which a total of 27 miRNA and 250 genes were shown as significantly correlated (see list in Supplementary Materials Table S2). The results showed a separation of miRNAs and correlated genes that can be summarized in four clusters: (i) a core of 22 miRNAs with a long distance from the center (range: from −0.945 to −0.731) in variate 2; (ii) a cluster of 168 genes negatively correlated to miRNA variate 1 and constituting gene variate 1; (iii) a small cluster of five miRNAs in variate 2 (range: from −032 to −0.201); (iv) a cluster of 82 genes negatively correlated to miRNA variate 2 and constituting gene variate 2 (see Supplementary Materials Table S2 for details about miRNAs and genes belonging to the four clusters). To improve our understanding of the connections between miRNAs and genes, a pair-wise similarity matrix was computed and displayed by CIM (Figure 3B).

### 3.4. Computational Integration of miRNAs and Genes by MAGIA 2

**Figure 4:**
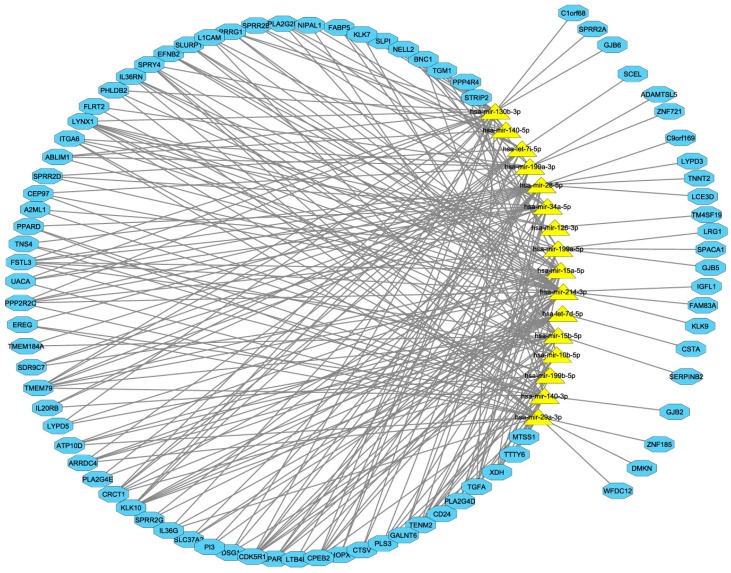
Computational integration of genes and miRNAs by MAGIA2 bioinformatics tool: The network of anticorrelated miRNA genes was computed by rCCA and integrated by MAGIA2 tools with the information of target prediction derived from DIANA-microT, TargetScan, and microrna.org. Two hundred and forty-five interactions were found among 16 miRNAs and 84 genes. The Cytoscape tool was applied to display the interconnections between features. Yellow triangles indicate the miRNAs, and blue boxes indicate the genes. If it is predicted that a gene is to be targeted by at least two out of 16 miRNAs, this gene is located in the central ring, comprising the miRNAs. Otherwise, it is placed outside.

The miRNA genes identified by rCCA were integrated for network analysis using three prediction target algorithms (DIANA-microT, TargetScan, microrna.org). By imposing a *q*-value < 0.1 and Pearson correlation as an association measure, 16 miRNAs and 84 genes were interconnected (Figure 4 and Supplementary Materials Table S3). Analysis of the 245 identified interactions highlighted that: (i) four miRNAs (hsa-miR-130b-3p, hsa-miR-199a-3p, hsa-miR-214-3p, and hsa-miR-28-5p) accounted for 41% of the interactions; (ii) four genes (CDK5R1, KLK10, LYNX1, and TMEM79) were co-targeted by eight miRNAs; (iii) several genes were targeted by several miRNAs; (iv) a survey using the miRTarBase repository proved that CD24/miR-34a-5p, ITGA6/miR-29a-3p, and L1CAM/miR-34a-5p were experimentally validated interactions.

### 3.5. Development of An Integrated miRNA-Gene Expression Predictive Model

**Figure 5:**
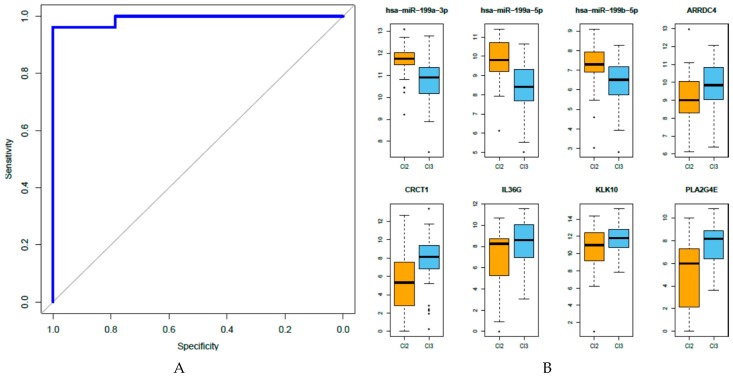
(**A**) Performance of the eight miRNA–gene expression predictive model: The performance of our signature (blue line) in predicting PFS after cetuximab-CT treatment was tested in terms of sensitivity and specificity by ROC analysis (AUC = 0.992); (**B**) Analysis of the expression of the eight features entered in the miRNA-gene integrated signature in HNSCC TCGA data set: The tumors were stratified based on our six HNSCC subtypes [34] and miRNA and gene expression data were retrieved for the Cl2-mesenchymal (30 samples) and Cl3-hypoxia (59 samples) subtypes. A significant upregulation of hsa-miR-199a-3p, hsa-miR-199a-5p, and hsa-miR-199b-5p in Cl2 (*p* = 7.25E-05, *p* = 3.8E-06, *p* = 0.0019, respectively) and of ARRDC4, CRCT1, IL36G, KLK10, and PLA2G4E in Cl3 (*p* = 0.021, *p* = 4.66E-05, *p* = 0.00199, *p* = 0.0135, *p* = 2.42E-06, respectively) was observed.

We investigated to what extent the miRNA-gene integrated network could predict the outcome in our cohort of patients. A feature selection was imposed by means of stSVM to the miRNA-gene networks identified by rCCA, along with the adjacency matrix representing the biological interactions found by at least two of the three target prediction algorithms applied; we expected to select the most relevant features for predicting the outcome. As such, the results highlighted eight relevant features (three miRNAs and five genes): hsa-miR-199a-3p, hsa-miR-199a-5p, hsa-miR-199b-5p, ARRDC4, CRCT1, IL36G, KLK10, and PLA2G4E. The performance of the identified signature, as assessed through Receiver Operator Characteristic (ROC) analysis, reached an AUC of 0.992 (Figure 5A).

### 3.6. Analysis of the Eight miRNA-Gene Integrated Signatures in TCGA Data

Our previous analysis of gene expression of the same patients [4] demonstrated that long- and short-PFS patients are characterized by prevalently belonging to HNSCC tumor subtypes Cl2-mesenchymal and Cl3-hypoxia, associated with long- and short-PFS, respectively. The utilized subtype stratification was previously identified using a wide meta-analysis of publically available gene expression HSNCC datasets and was validated in other datasets including TCGA [34]. Since TCGA provides both miRNA and gene expression profiles, we investigated whether the three miRNAs and the five genes entered in the integrated signature could be associated with the Cl2 and Cl3 subtypes. The TCGA gene expression profile was used for subtype stratification, resulting in 30 and 59 samples classified as Cl2 and Cl3, respectively. These two sets of TCGA samples were used to analyze the expression of the eight features of our predictive signatures; in agreement with our hypothesis, a significant upregulation of the three miRNAs (hsa-miR-199a-5p, hsa-miR-199a-3p, and hsa-miR-199b-5p) was observed in the Cl2-mesenchymal subset of samples, while a significant upregulation of the five genes (ARRDC4, CRCT1, IL36G, KLK10, and PLA2G4E) was found in the Cl3-hypoxia subset of samples (Figure 5B).

## 4. Discussion

Combining data from multiple sources has the potential to draw a more comprehensive view of the biological systems, eventually enabling an improved prediction performance [35]. To our knowledge, the integrative analysis of miRNA and mRNA expression presented here is the first attempt on a cohort of HNSCC patients treated with EGFR inhibitors. Overall, using a well-defined clinical setting of pretreatment recurrent/metastatic HNSCC FFPE specimens and up-to-date bioinformatics tools, we demonstrated the proof of principle that an integrative miRNA-mRNA expression analysis could greatly contribute to a refinement in the identification of the biology behind the response to palliative cetuximab-based treatment and to the development of a potential predictive model.

High-throughput technologies have proved to be of great potential for gaining valuable insights into tumor biology, and even to build predictive models in HNSCC [36]. However, these data involve a number of variables (i.e., miRNA, genes), by far over-exceeding the number of samples, resulting in a high degree of multicollinearity and ill-conditioned issues. Many bioinformatics pipelines have been developed to solve these issues, mainly reducing the dimensionality of the data [37]. Taking into account a supervised classification setting, the approach involves the introduction of a new artificial variable able to summarize most of the information present in highly correlated miRNAs or genes. In this context, sparse partial–least squares discriminant analysis (sPLS-DA) is a well-known, supervised, pattern-recognition method able to unravel the information contained in a data matrix in relation to a qualitative response variable indicating classes of samples [10].

Long- and short-PFS cases were compared on the basis of their miRNA profiles using sPLS-DA. Our results clearly indicate that miRNA profiles separated the two groups of patients well, and the lowest misclassification rate was obtained considering a total of 36 miRNAs in two components. When this signature was compared to a supervised class comparison, most of the miRNAs differentially expressed by a random variance *t*-test–based class comparison were included in the first component and none in the second.

In general, miRNA expression profiles provide limited information about their biological role. In fact, miRNAs being non-coding regulators of gene expression, their function is linked not only to their expression level, but also to those of their target genes. In this context, an integrative analysis could help uncover the biological regulatory interactions between miRNAs and gene expression.

First of all, we investigated the association of miRNA sPLS-DA with gene expression data. A significant correlation between first-component miRNAs and pathways enriched in long-PFS patients (EGFR signaling, keratinocyte differentiation, ectoderm development pathways and p53 and Myc oncogenic signatures) recapitulates our previous observations obtained by directly analyzing gene expression [4], but also identified the association with hypoxia. An activated EGFR pathway leads to a response to cetuximab. In this context, CD24, a mucin-like membrane glycoprotein, regulates the expression of EGFR by preventing its internalization and RhoA-dependent degradation [38]. Our data clearly demonstrated an interaction between hsa-miR-34a-5p and its experimentally validated target CD24, as hsa-miR-34a-5p was downregulated while CD24 was upregulated in long-PFS patients. The combination of platinum-based chemotherapy and targeted therapy with an anti-EGFR inhibitor represents a strategy in treating RM-HNSCC. However, little is known about the mechanism leading to chemo-resistance [39]. It has been demonstrated that inhibition of L1 cell adhesion molecule (L1CAM) and EGFR sensitizes cells to cisplatin [40] and our findings support a molecular loop among EGFR signaling activation, the downregulation of hsa-miR-34a-5p and the upregulation of its L1CAM target in long-PFS patients.

Through a meta-analysis approach of gene expression profiling data, we identified subtypes with distinct molecular features including subtypes over-expressing hypoxia and mesenchymal pathways [34]. By association with drug sensitivity, the hypoxia subtype shows the greatest sensitivity to EGFR inhibitors [34]. In Bossi et al. [4], in a series of cetuximab-treated patients, we confirmed a molecular link between long-PFS patients and the Cl3-hypoxia subtype, and between short-PFS patients and the Cl2-mesenchymal subtype. Similar results were obtained following Keck’s subtype stratification [41]. In addition, our integrated analysis disclosed the interplay between ITGA6, a hypoxia-regulated gene [42], and its experimentally validated target hsa-miR-29a-3p. Remarkably, the miRNA sPLS-DA second component, associated with short-PFS, confirmed the role of the RAS pathway [4], and the results mainly correlated with an immune/inflammatory response hub that comprises eight functionally related networks (immune response, inflammatory response, defense response, cytokine production, TNFA signaling, interferon gamma, interleukin, and leukocyte activation). In addition, inferring the gene networks associated with the miRNA sPLS-DA second component, we noticed a significant activation and enrichment of the epithelial–mesenchymal transition, angiogenesis, and NOTCH pathways, in agreement with their involvement in the Cl2-mesenchymal subtype [34]. Overall, our data strongly suggests that the miRNA sPLS-DA not only supports the biology previously identified as associated with long and short PFS [4], but provides new evidence. In particular, the sPLS-DA second component provides a completely new layer of information, not captured by differential expression analysis which strongly contributed to improving our knowledge about the biology behind the response to cetuximab-CT, and could open the way to new therapeutic suggestions. Anti–PD-1 therapies were reported to evade the checkpoint blockade, resulting in the reactivation of the immune system, enabling the eradication of the host tumor and improving, eventually, patient survival [43,44]. The significant enrichment of the immune/inflammatory pathway might suggest a potential benefit to immune therapy in short-PFS patients.

In systems biology, a major challenge in the integration effort of multi-omics and highly dimensional data is the extraction of meaningful information. Since it is expected that similar expression patterns across a set of samples potentially have a functional relationship, we applied a regularized version of canonical correlation analysis (rCCA) to explore correlation structures between miRNA and gene quantitative features assessed on the same cohort of patients [20]. Exploratory approaches with correlation circle plots and clustered image maps highlighted the association between 27 miRNAs and 250 genes, proving the existence of distinct anti-correlated expression patterns. These associations were confirmed by target prediction algorithms and in more detail. As such, all four members of the miR-199/214 cluster, and five out of the 12 members of the let-7 family, participate in the miRNA-mRNA network; a regulatory hub defined by four miRNAs (hsa-miR-130b-3p, hsa-miR-199a-3p, hsa-miR-214-3p, and hsa-miR-28-5p) and their mRNA targets, involving 41% of identified interactions, was observed.

Focusing our attention on the miRNA-gene integrated features able to stratify patients according to their response to cetuximab-based treatment, a signature based on three miRNAs and five genes was identified. The height miRNA-gene integrated signature showed an excellent accuracy in predicting treatment response (AUC = 0.992), while the single features reached an AUC ranging from 0.85 to 0.90. It was noteworthy that all three miRNAs belonged to the miR-199/214 cluster, and all five genes were regulated by two members of the let7 family (let7d and let7i). These data, together with the observation that when their expression was tested in HNSCC TCGA, the three miRNAs were upregulated in the Cl2-mesenchymal subtype, while the five genes were upregulated in the Cl3-hypoxia subtype, strongly support the hypothesis that these miRNAs and their regulated genes have a pivotal role in HSNCC progression and sensitivity to therapy.

We are aware that miRNAs, by binding to mRNA molecules, can inhibit their translation or induce degradation. However, most of the available tools and methods for miRNA target prediction, such as those here applied, have been developed following the assumption of an inverse correlation between miRNA/mRNA and they tend to miss the targets where expression repression is caused by translation repression. Since in the future we will expect a strong improvement in the knowledge on miRNA biology, the public availability of our miRNA and gene expression data for the scientific community at the GEO website will enable any future analysis of the interplay between miRNAs and genes.

## 5. Conclusions

Overall, through gene and miRNA expression profiling of the same samples of HNSCC and an integrative miRNA-mRNA expression analysis, we identified candidate biomarkers of PFS in patients treated with cetuximab-based approaches. The use of a well-defined clinical settings and up-to-date bioinformatics tools enabled to give the proof of principle that an integrative miRNA–mRNA expression could greatly contribute to further gain insight into the biology and prediction, and could have potential direct implications on clinical care. Since we selected patients with marked opposite outcomes after treatment, to transpose our predictor of response into a useful clinical grade assay, additional work is needed.

## Supplementary Material

The following are available online at www.mdpi.com/2073-4425/8/1/35/s1. Figure S1: Tuning of parameters for rCCA, Figure S2: Volcano plot for the 614 miRNA detected by microarray analysis, Figure S3: Gene expression sPLS-DA in patients treated with cetuximab, Table S1: miRNA identified by supervised analysis using sPLS-DA and by supervised class comparison analysis, Table S2: List of 27 miRNA and 250 genes identified by rCCA, Table S3: List of the 245 interactions disclosed by MAGIA2 tool.
