# Supplementary material for: Integrative miRNA-Gene Expression Analysis Enables Refinement of Associated Biology and Prediction of Response to Cetuximab in Head and Neck Squamous Cell Cancer
Source: Genes (Basel). 2017 Jan 14;8(1):35. doi: 10.3390/genes8010035 (PMC5295029; doi:10.3390/genes8010035)
Supplement: Supplementary file 1 [file genes-08-00035-s001.zip › supplementary/genes-158636-suppl.-final.docx]

Supplementary Materials: Integrative miRNA-Gene Expression Analysis Enables Refinement of Associated Biology and Prediction of Response to Cetuximab in Head and Neck Squamous Cell Cancer

Loris De Cecco, Marco Giannoccaro, Edoardo Marchesi, Paolo Bossi, Federica Favales, Laura D. Locati, Lisa Licitra, Silvana Pilotti and Silvana Canevari

| 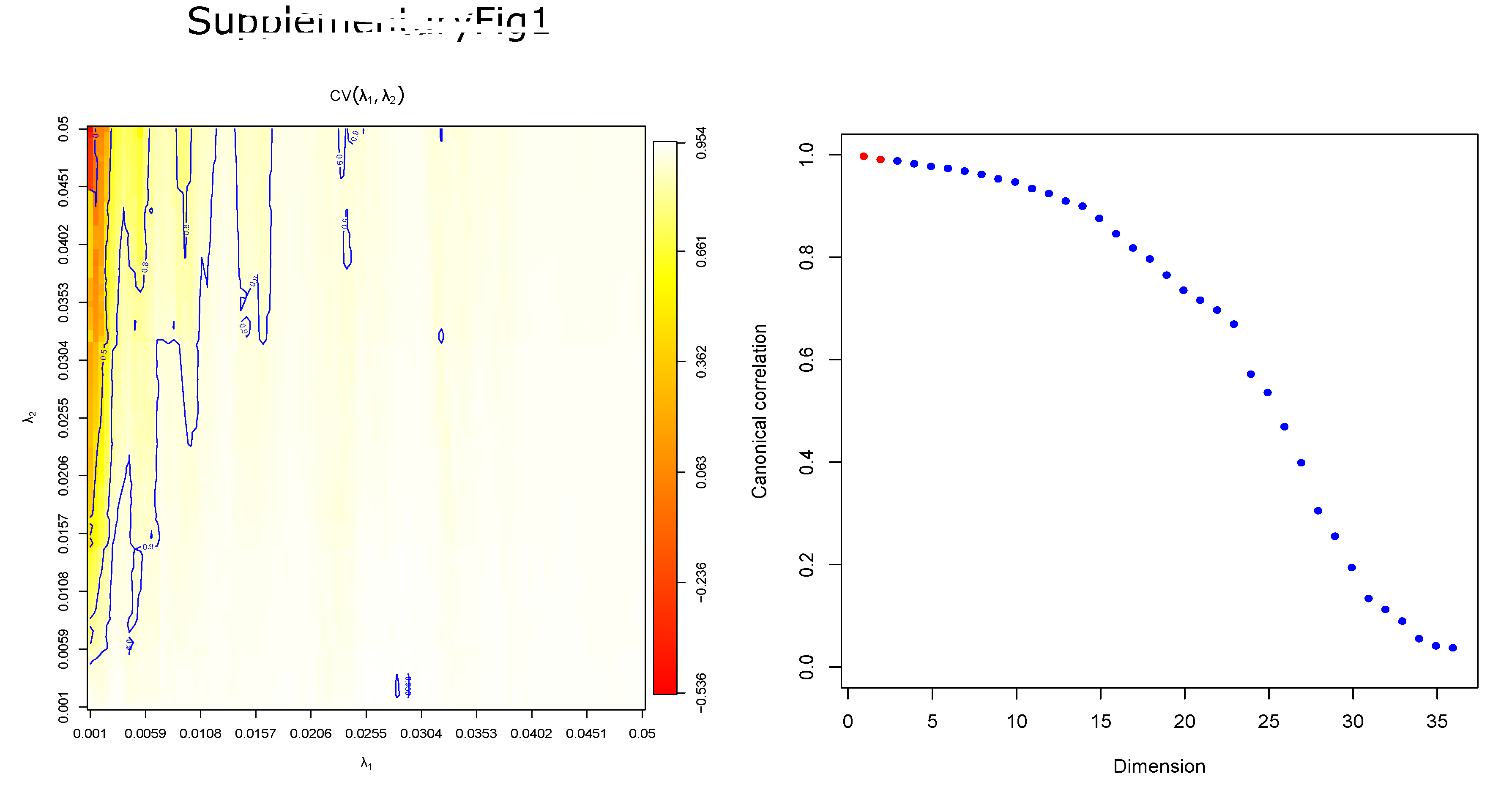 | 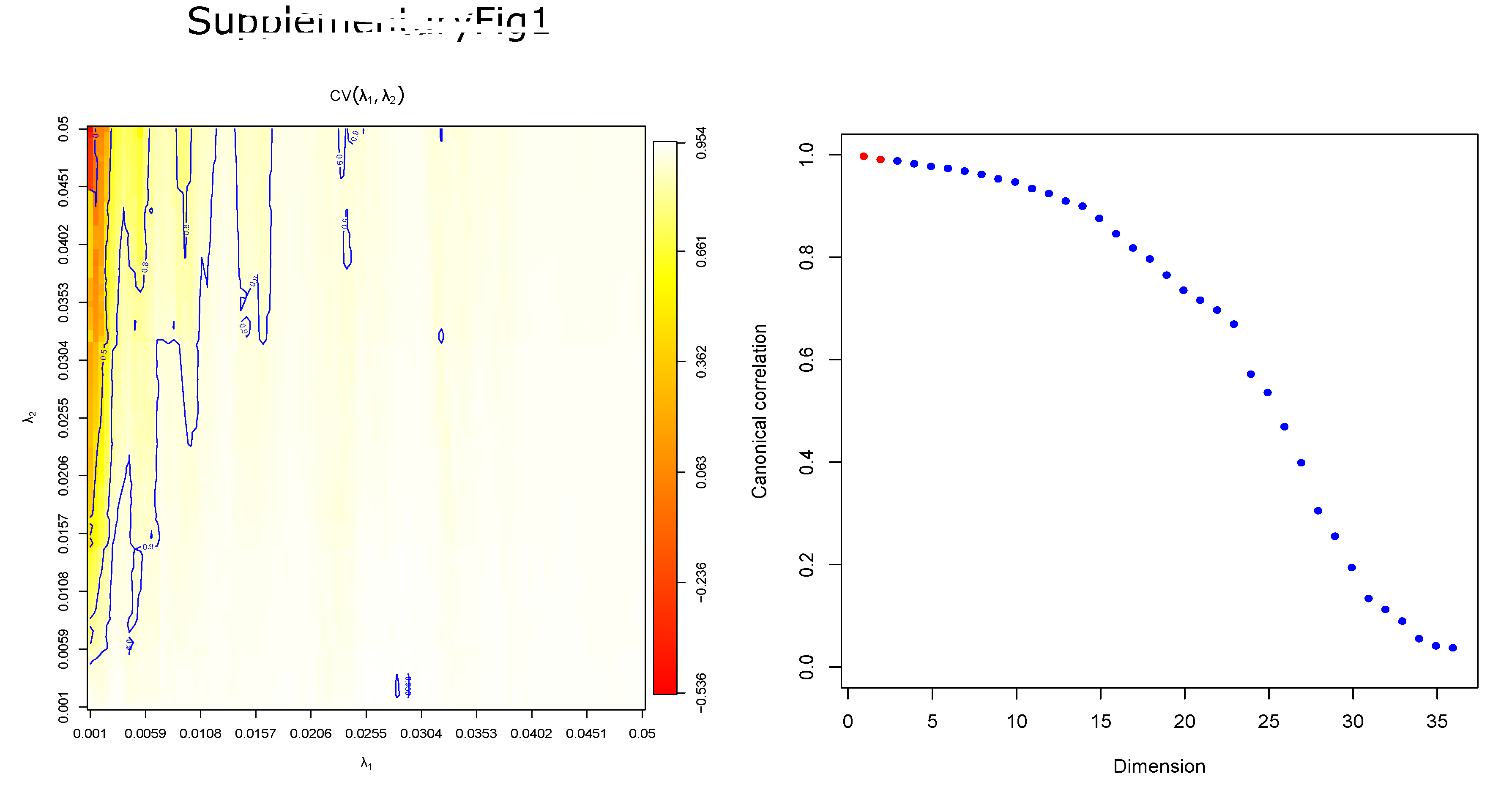 |
| --- | --- |
| A | B |

**Figure S1.** Tuning of parameters for rCCA: (**A**) The plot CV (λ1, λ2) displays the point-by-point CV-score grid. The regularization parameters λ1 and λ2 were selected by 10-fold cross-validation procedure based on a grid of equally-spaced discretization values of size 100 × 100 spanning an interval of 0.001 < λ1, λ2 < 0.05. For values equal to (0.5-0.8-0.9-0.954) a contour plot was added in blue and with a value of 0.956 the CV (λ1, λ2) reached its maximum correspondence at λ1 = 0.0292 and λ2 = 0.00199; (**B**) In the screen graph for the 36 canonical correlations imposing the obtained λ1 and λ2 parameters, a clear gap was observed between the 23rd and 24th dimensions. The first 23 variates were investigated for their capability to explain most data variability. Since the first two variates represent 59% of data variability, they were chosen for final representation and analysis of rCCA (thick red points).


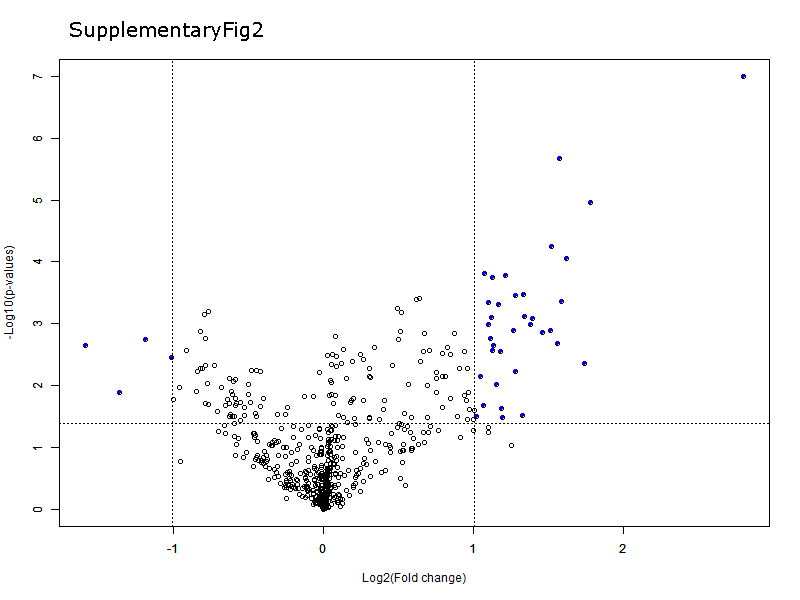


**Figure S2.** Volcano plot for the 614 miRNA detected by microarray analysis: The X-axis represents the log_2_ (fold-change), and the Y-axis represents the −log_10_ (*p*-value). A total of 39 miRNAs (blue dots) were expressed differentially, imposing an FDR < 0.15 and a |log_2_ (fold change)| > 1.

| 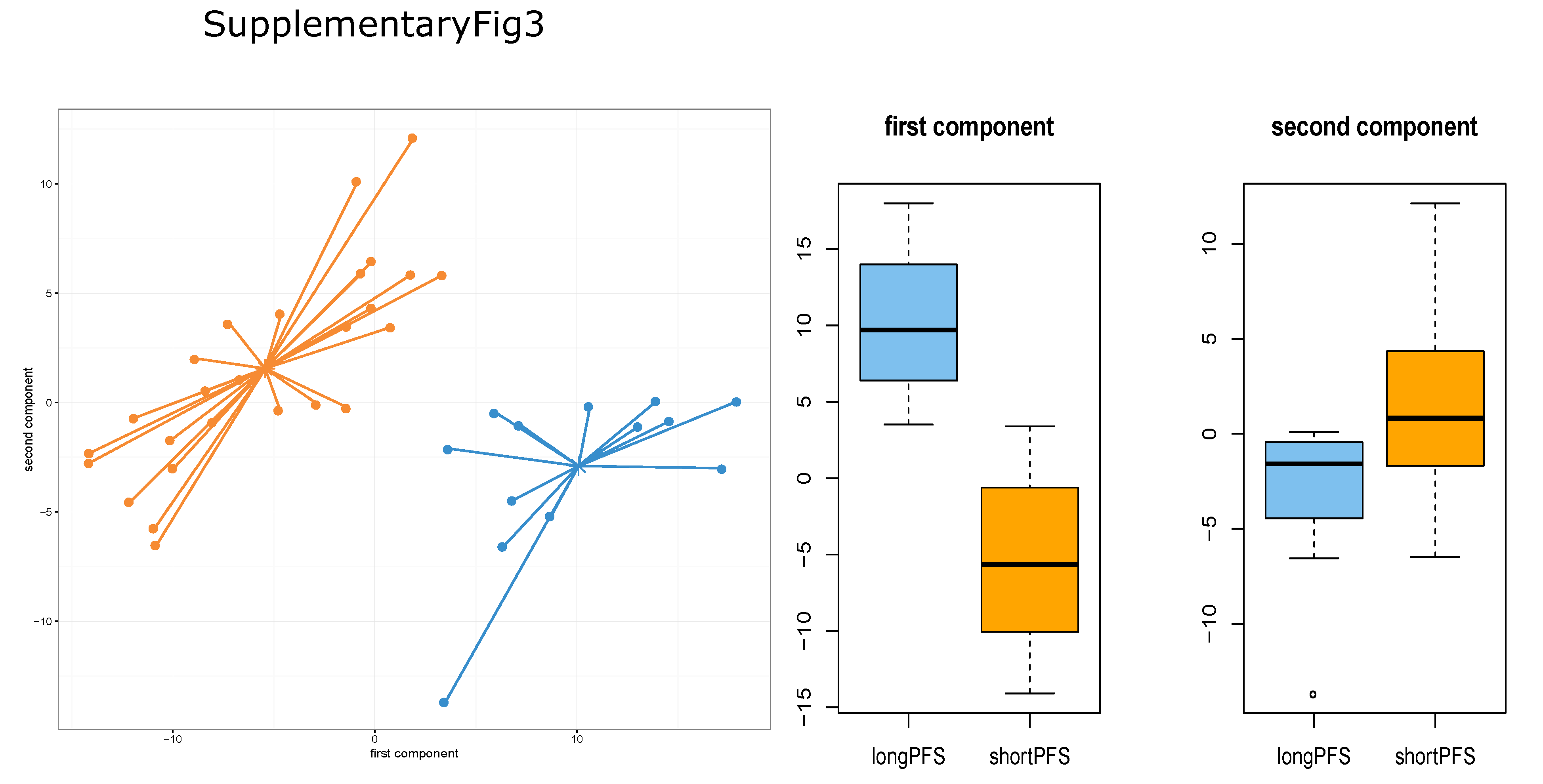 | 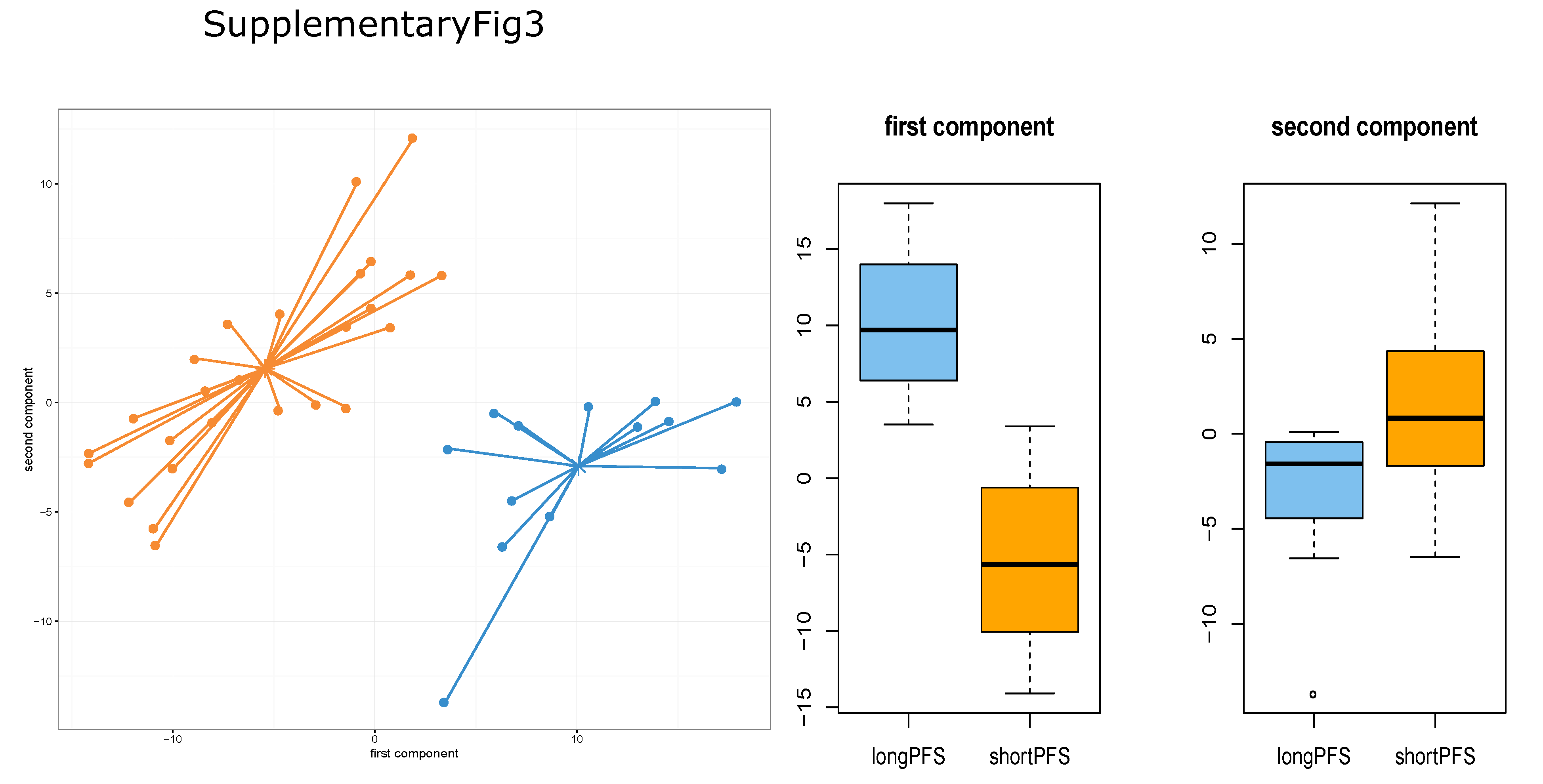 |
| --- | --- |
| A | B |

**Figure S3.** Gene-expression sPLS-DA in patients treated with cetuximab. (**A**) The score plot of the first two components is shown for each individual patient. Two well-defined clusters can be identified corresponding to 14 long-PFS patients (blue dots) and 26 short-PFS patients (orange dots). The lines indicate the distance of each sample to the centroid for the two classes; (**B**) The box-plots show the loading vector values for first component and second component with samples divided, based on the response to treatment. A significant difference of *p* = 5.97E-11 and *p* = 0.00359 in the first component and second component, respectively, was observed.

© 2017 by the authors. Submitted for possible open access publication under the
terms and conditions of the Creative Commons Attribution (CC-BY) license (http://creativecommons.org/licenses/by/4.0/).
